# A Cross-Sectional Analysis of Paternal Intimacy Problems, Stress Levels, and Satisfaction from Families with Children Born with Mucoviscidosis

**DOI:** 10.3390/ijerph192215055

**Published:** 2022-11-16

**Authors:** Zoran Laurentiu Popa, Madalin-Marius Margan, Elena Bernad, Lavinia Stelea, Marius Craina, Ioana Mihaela Ciuca, Anca Mihaela Bina

**Affiliations:** 1 Department of Obstetrics and Gynecology, “Victor Babes” University of Medicine and Pharmacy, 300041 Timisoara, Romania; 2 Centre for Translational Research and Systems Medicine, “Victor Babes” University of Medicine and Pharmacy, 300041 Timisoara, Romania; 3 Department of Microscopic Morphology, “Victor Babes” University of Medicine and Pharmacy, 300041 Timisoara, Romania; 4 Department of Pediatrics, “Victor Babes” University of Medicine and Pharmacy, 300041 Timisoara, Romania; 5 Department III Functional Sciences, Discipline of Pathophysiology, “Victor Babes” University of Medicine and Pharmacy, 300041 Timisoara, Romania

**Keywords:** cystic fibrosis, mucoviscidosis, paternal stress, marital attitudes, sexual dysfunction

## Abstract

There is an increasing interest in father–child interactions and their effects. Due to the rising number of working mothers, marital interruptions, divorces, and child custody arrangements, paternal duties and the relevance of fathering continue to be re-evaluated. As there are rising expectations for men to undertake more childcare and household responsibilities, it was hypothesized that the presence of a disabled or chronically ill child would have a significant impact on the couple’s future family situation, marital conduct due to paternal dissatisfaction, and increased stress levels. Therefore, the purpose of this study was to examine paternal intimacy problems, stress levels, and couple satisfaction inside families that have children with cystic fibrosis. The study followed a cross-sectional design with five questionnaires that were answered by a total of 107 fathers of children with cystic fibrosis from the “cases” group as the reference group, and 124 fathers of healthy children from the “control” group. The statistically significant findings of the current study show that men who were taking care of their child with mucoviscidosis engaged less frequently in sexual activity. A significantly higher number of these respondents were smokers. A higher proportion of them reported marital distress (OR = 2.54) and inhibited sexual desire (OR = 2.02), all in association with a higher number of men taking psychiatric medication (7.5% vs. 1.6%). More than 40% of all respondents declared high levels of general stress and parenting distress, while the most frequently used coping mechanism for stress was avoidance-oriented (45.8% vs. 25.8%). Other important findings were the high levels of dissatisfaction and lower levels of marital quality on the SII scale, equivalent to the intimacy problems on the MIQ scale. It is likely that paternal stress is higher when parenting children with cystic fibrosis, and the lack of intervention in this vulnerable group seem to be associated with intimacy problems, couple dissatisfaction, and maladaptive coping mechanisms. It is recommended that these concerns should not only be raised for the mothers of children with mucoviscidosis, but also for the child’s father or the male caretaker partner since they might experience the same problems as the opposite gender.

## 1. Introduction

Mucoviscidosis, often known as cystic fibrosis (CF), is the most frequent significant genetic illness seen in Caucasian populations [1], and since its discovery, it has been considered to affect only children due to its very low life expectancy. Seventy years ago, those affected would live, on average, only two years. The disease is caused by a mutation in the CFTR gene and may cause clinical symptoms in a variety of systems, including the respiratory, sinus, digestive, and reproductive tracts. Monitoring pulmonary function, as well as nutritional status, airway clearance, and infection management, is an integral part of standard medical practice at present [2]. With the increase in treatment capabilities and management options a new demographic is forming, which consists of children, adolescents, and adults who have cystic fibrosis [3,4,5].

There is evidence to suggest that parents and caregivers of children who are diagnosed with a chronic disease report considerably greater levels of general parenting stress than caregivers of normally developed children [6,7]. In addition, parental stress is an essential component in making sense of any dysfunction or psychopathology that may exist in the family. On the other hand, it is not yet known to what degree parental stress is a prominent aspect of a family with a child suffering from cystic fibrosis, as not all surveys indicate an increased level of stress or how this stress can affect the well-being of the family [8]. As consequence of increasing overall stress levels inside the family, specific problems might arise that can form a vicious circle, such as decreased couple satisfaction levels, intimacy problems, and sexual dysfunction.

Besides the existing literature studying maternal behavior in a family with children suffering from a chronic illness [9], researching paternal attitudes is also important for a number of compelling reasons, both in the real world and in the academic world. A better knowledge of the ways in which fathers cope with such stressful situations and the way they affect their physical and mental health may be helpful to those who provide supporting services in their attempts to harness the strengths of families and counteract the consequences of prolonged stress [10]. Recent shifts in the traditional roles of the family structure and parents have increased the significance of the paternal difficulties [11], as both mothers and fathers now share nearly complete responsibility for the caretaking of their children, with very little aid from surrogate caregivers in the family. It seems that men with children are taking on a bigger role in the care of their offspring as a result of contemporaneous developments, most notably the increased resolve among a significant number of mothers to continue employment outside of the household. As a consequence, there is a growing possibility that fathers may experience the challenges faced by their children in a more direct and meaningful way [12].

Because the mental health of caregivers is seldom taken into account throughout the course of therapy for pediatric patients, it has been considered interesting to determine the level of stress experienced by parents of children who have been diagnosed with mucoviscidosis, and how these parents may feel in light of both the physical and emotional commitments that are required of them in caring for their child. This is because perceived stress while parenting is a common part of the experience of being a parent, and it typically occurs when one or both parents’ commitments exceed their own internal resources [13]. Therefore, this research hypothesized that intimacy problems and stress levels are likely to increase among families with a child suffering from cystic fibrosis. Due to a reduced research focus, the aim of this paper was to elucidate specifically the ways in which fathers are impacted psychologically by having a child with mucoviscidosis and how the presence of a child with cystic fibrosis affects paternal intimacy, stress levels, and marital satisfaction.

## 2. Materials and Methods

### 2.1. Study Design and Participants

The current research was designed as cross-sectional, survey-type research, at the Victor Babes University of Medicine and Pharmacy in Timisoara, Romania. The distribution of questionnaires and data collection was completed between January 2022 and June 2022. The study participants were men older than 18 years whose children with cystic fibrosis were born at the University Clinic of Obstetrics and Gynecology “Bega”. Patients were provided with information on the objectives and potential outcomes of the research, and in order to participate in the study, each patient was required to sign a written informed consent form. The questionnaires were administered online in compliance with the pandemic constraints imposed by the SARS-CoV-2 virus. Data collection was carried out on the basis of the full responses to the questionnaires provided and answers that were obtained in conjunction with the paper records of couples who were followed at our clinic. Incomplete patient consent forms, surveys, and paper records were all taken into consideration as potential exclusion factors. Our study was carried out in accordance with the principles outlined in the Helsinki Declaration concerning the conduct of scientific research involving volunteers from the general public, and it was given approval by the Scientific Ethics Committee of the Timisoara Clinical Emergency Hospital “Pius Brinzeu” on 6 June 2022, under the approval number 303.

### 2.2. Study Instruments

To assess paternal intimacy problems, stress levels, and couple satisfaction when taking care of a child with cystic fibrosis, we selected only men who had a child with mucoviscidosis born in our clinic. In order to determine the proper sample size for the group of males coming from families with a child suffering from cystic fibrosis, a technique of convenience sampling was employed. With a margin of error of 1% at a confidence level of 99% and an assumed frequency of mucoviscidosis of less than 1% in the general population, it was estimated that the sample would suffice to consist of at least 67 men. The sample size calculation was based on epidemiology features of the disease, and the assumption was based on the fact that the overall prevalence of mucoviscidosis is less than 1% [14]. Of 150 fathers who agreed to complete the surveys, there were 107 complete answers returned at the end of the study period that were included in the analysis as the “cases” group—the study reference group. Another comparison group comprising an equivalent number of respondents with similar characteristics was requested to complete the same surveys as the “control” group. The control group was considered to include fathers of healthy children, with the same age characteristics as the reference group. There were 124 complete responses included in the analysis from the control group. All missing data (69 respondents) were removed due to incomplete questionnaires.

The participants were asked questions on their demographics, history of depression, stress levels, quality of life, coping techniques, and couple satisfaction. The following five standardized questionnaires, translated into the Romanian language, were given to the participants: (1) Coping Inventory for Stressful Situations (CISS); (2) Parenting Stress Index—Short Form (PSI-SF); (3) Sexual Interaction Inventory—(SII); (4) the Dyadic Sexual Communication Scale (DSCS); and (5) the Marital Intimacy Questionnaire (MIQ).

The Coping Inventory for Stressful Situations (CISS) is a tool that evaluates different ways of coping with stressful situations [15]. Using a scale similar to the Likert scale, its items address a variety of facets of human behavior as statements that are either negative or positive. The scale ranges from 1 (never) to 6 (very often). The CISS gives three scores that are task-oriented, emotion-oriented, and avoidance-oriented. Task-oriented refers to an individual’s focus on completing a task. There are sixteen components that make up each subscale. Scores that are higher on the scale reflect a greater level of intensity in the coping mechanism.

The Parenting Stress Index—Short Form (PSI-SF) is a standardized questionnaire that can be completed by parents without any particular guidance and is frequently used in clinical practice. It was developed by the National Institute of Child Health and Human Development [16], and it assesses parental and child-related stress, as well as the environment in which the interaction takes place. The PSI-SF comprised a total of 36 questions, each of which was graded using the Likert scale. The responses describe the degree to which individuals agreed or disagreed with the proposition that was offered. The levels of agreement are as follows: strongly agree, agree, not sure, disagree, and strongly disagree. In addition to this, the questionnaire was broken up into three sections, which were as follows: (1) Parenting Distress—PD; (2) Parent–Child Dysfunctional Interaction—P-CDI; and (3) Difficult Child (DC). High stress was defined as a stress level that fell between the 85th and 100th percentiles and was regarded to be clinically relevant and labeled as high.

The Sexual Interaction Instrument, sometimes known as the SII, is a self-report inventory that is used to evaluate many elements of heterosexual couples’ sexual experience [17]. The Sexual Interest Inventory comprises a list of 17 different sexual activities. When evaluating each action, the husband or the wife responds to a set of six questions that are scored using a scale with six points. The results of each partner’s participation in 17 activities are added together, and the combined totals are utilized to create a profile consisting of 11 different subscales. Male self-acceptance, male enjoyment mean, male perceptual accuracy of females, and male acceptance of females are the subscales that make up the male frequency of discontent.

The Dyadic Sexual Communication Scale, often known as the DSCS, is also a Likert scale that investigates how respondents feel about the communication process that is involved in sexual relationships. Items are rated on a six-point scale, with one representing strong agreement and six representing significant disagreement. The scores of the sample on the DSCS scale range from 15 to 78. Higher scores on DSCS are indicative of better perceived sexual communication between spouses, as seen from the male perspective [18].

The Marital Intimacy Questionnaire (MIQ) consists of 56 questions, each of which is assessed using a Likert scale that ranges from 1 (not at all true) to 5 (very true). Scores are derived from the Multidimensional Intimacy Questionnaire (MIQ) based on five aspects of intimacy: intimacy difficulties, consensus, openness, love, and commitment. The higher the score on the intimacy problem subscale, the more significant the issue is in that domain. When it comes to intimacy, having higher scores on the other four subscales indicates greater functioning in each of those areas [19].

### 2.3. Statistical Analysis

IBM SPSS Statistics.27.0, as well as Microsoft Excel [20,21], were used to conduct descriptive and inferential statistical analysis between the two main study groups of cases from families with cystic fibrosis children, and the controls from families with healthy children. For the purpose of describing continuous and numerical data regarding survey scores, we used the mean and standard deviation; however, the categorical variables were represented by absolute values and percentages. For this inquiry, we compared the mean values of the surveyed scores that were normally distributed by using the Student’s *t*-test. In order to carry out comparisons of proportions (%) between the two study groups, we employed the Chi-square test. It was decided that an alpha equal to 0.05 would serve as the significance level.

## 3. Results

### 3.1. Patients’ Background

The two comparison groups comprised 107 cases represented by men who are fathers of a child with mucoviscidosis, and 124 controls represented by fathers who have children without disabilities and genetic disorders. The comparison of background characteristics presented in Table 1 indicated no significant differences between cases and controls regarding their age distribution, area of residence, relationship status, education, income, occupation, or religion. However, it was observed that the differences reported in regard to sexual activity were statistically significant between groups. There were 37.4% of male participants who admitted to engaging in sexual activity less than 1–3 times per month, compared with 21.0% in the control group of men who are fathers of children without disabilities (*p*-value = 0.021). Moreover, it was observed that 34.6% of men with children who suffer from cystic fibrosis reported smoking frequently, compared with only 22.6% in the control group (*p*-value = 0.043). This significant difference is likely attributed to smoking being a stress-relief method for caregivers of sick children.

### 3.2. Unstandardized Survey Questions

The comparison of unstandardized survey questions between cases and controls described in Table 2 showed that couples from families where a child with mucoviscidosis was born were more likely to have fewer children, compared to the normal families where 38.7% had two or more children (*p*-value < 0.001). The age distribution of their children was significantly different, with 10.3% adolescents in the cases group, compared with 24.2% adolescents in the control group. The proportion of self-reported marital distress was also significantly increased in the group of fathers with children suffering from cystic fibrosis (15.0% vs. 6.5%, *p*-value = 0.034), with an odds ratio (OR) of 2.54, indicating a 2.54 times higher likelihood of marital distress in this group. It was also observed that more men in the cases group reported the use of psychiatric medication (7.5% vs. 1.6%, *p*-value = 0.028). The computed odds ratio was 4.92, indicating a 4.92 higher likelihood for men in this category to be under psychiatric medication. Lastly, the respondents from the cases group had a significantly increased inhibition of sexual desire (24.3% vs. 13.7%, *p*-value = 0.039), with a 2.02 times higher likelihood (OR = 2.02), although this might be partially caused by the use of psychiatric medication such as selective serotonin reuptake inhibitors.

### 3.3. Analysis of Standardized Questionnaires

**Figure 1:**
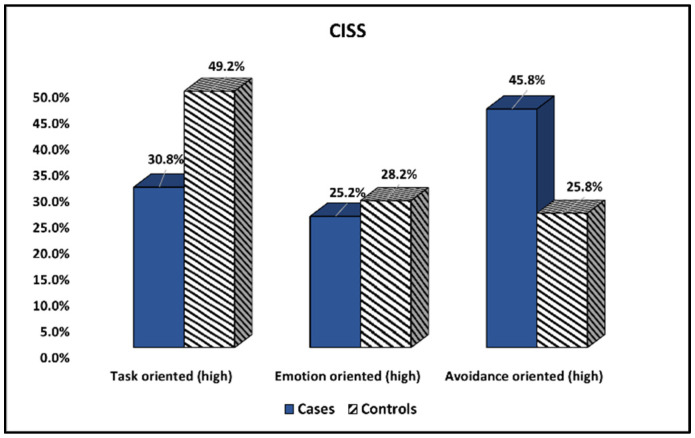
Comparison of CISS survey results between cases and controls.

The Coping Inventory for Stressful Situations (CISS) survey results compared between cases and controls are presented in Table 3 and Figure 1. It was observed that fathers with healthy children score significantly higher on the task-oriented coping method (49.2% vs. 30.8%, *p*-value = 0.004). The emotion-oriented coping mechanism did not differ between the two study groups; however, men who were fathering children with cystic fibrosis were significantly more likely to express an avoidance-type coping style (45.8% vs. 25.8%, *p*-value = 0.002).

**Figure 2:**
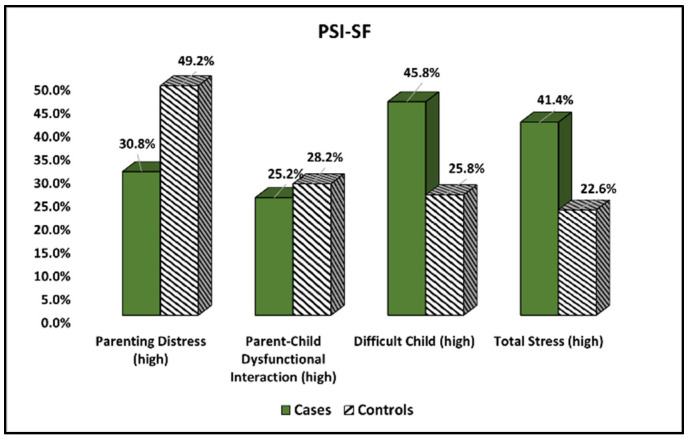
Comparison of PSI-SF survey results between cases and controls.

The Parenting Stress Index—Short Form (PSI-SF) results compared between cases and controls are presented in Table 4 and Figure 2. It was observed that parenting distress was experienced more often in a family with children suffering from mucoviscidosis (43.0% vs. 26.6%, *p*-value = 0.008). Other significant differences were observed in the difficult child inventory and total stress levels (34.6% vs. 18.5%, *p*-value = 0.005), respectively (41.4% vs. 22.6%, *p*-value = 0.002).

**Figure 3:**
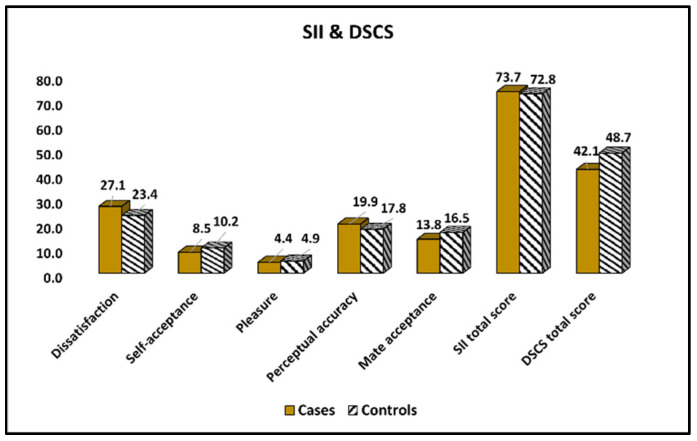
Comparison of SII survey results between cases and controls.

The Sexual Interaction Instrument (SII) and the Dyadic Sexual Communication Scale (DSCS) results compared between cases and controls are presented in Table 5 and Figure 3. It was observed that the mean levels of dissatisfaction were significantly higher in the cases group (27.1 vs. 23.4, *p*-value < 0.001). On the other side, the respondents with healthy children reported higher levels of self-acceptance and marital quality on the sexual interaction instrument scale, as well as significantly higher DSCS total scores (48.7 vs. 42.1, *p*-value < 0.001).

**Figure 4:**
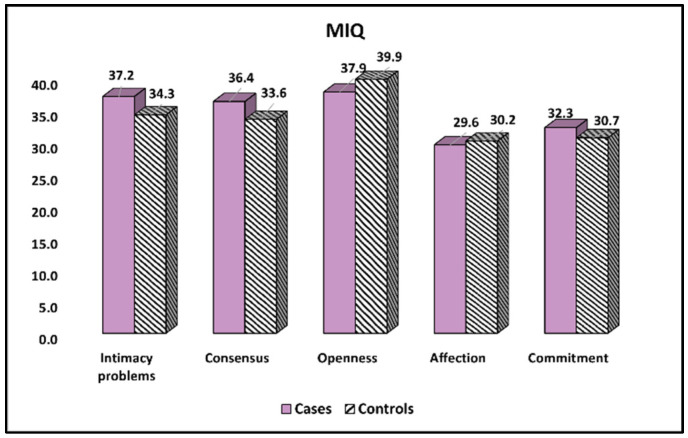
Comparison of MIQ survey results between cases and controls.

Lastly, The Marital Intimacy Questionnaire (MIQ) results are described in Table 6 and Figure 4. It was observed that intimacy and consensus problems were more often experienced by fathers of children with cystic fibrosis (37.2 vs. 34.3, *p*-value = 0.021), respectively (36.4 vs. 33.6, *p*-value = 0.004). On the contrary, commitment levels were observed to be significantly higher in the control group (32.3 vs. 30.7, *p*-value = 0.047).

## 4. Discussion

### 4.1. Literature Analysis

This research identified important findings in understanding how paternal attitudes, perceptions over couple satisfaction, intimacy problems, and stress levels correlate when there is a child with cystic fibrosis who is born inside the family. Among the significant findings in these respondents, it was observed that they engaged less frequently in sexual activity. It was also observed that the cases group had a significantly higher proportion of smokers. Although smoking parents are more likely to have an unhealthy child, and smoking was proven to cause CFTR dysfunction that is involved in cystic fibrosis, no direct causality was linked between paternal smoking and cystic fibrosis in children [22]. Additionally, a higher proportion of them reported marital distress and inhibited sexual desire, all in association with a higher number of men taking psychiatric medication. More than 40% of all respondents declared high levels of general stress and parenting distress, while the most frequently used coping mechanism for stress was avoidance-oriented. Other important findings were the high levels of dissatisfaction and lower levels of marital quality on the SII scale, equivalent to the intimacy problems on the MIQ scale.

Parenting is inextricably linked to the presence of stress resulting from a variety of normal and abnormal life situations. Parenting a child with a disability may bring particular obstacles to the family’s ability to function, despite the fact that the majority of children cause mothers and fathers to experience stress [23]. It is estimated that 17% of children under the age of 18 in the United States have developmental impairments [24]. The authors describe fathers of children with disabilities as reporting considerably higher levels of parental stress than parents of healthy children [25]. Families with children who have educational challenges generally feel elevated levels of stress, particularly in regard to parental obligations. In a matched sample comparing parents of normally developing children with parents of children with Down syndrome, it was observed that parents of children with Down syndrome reported greater levels of parent-related stress, challenges in perceived parenting competence, health concerns, and parental depression [26].

Similarly, in the present research, the percentage of fathers with children suffering from cystic fibrosis who self-reported marital difficulty was considerably greater (15.0% vs. 6.5%), with an odds ratio (OR) of 2.54, suggesting a 2.54-fold increased chance of marital distress in this group. Additionally, 5.9% more fathers in the cases group reported using psychiatric medication. The estimated odds ratio was 4.92, showing a 4.92 times greater risk for males in this group to be on psychiatric medication, which may be related to a higher frequency of depression among parents of children with chronic conditions. Lastly, it was observed that the same respondents in the cases group had a 10.6% higher inhibition of sexual desire than fathers in the control group, with a 2.02 times higher odds ratio (OR = 2.02), although this may be partially attributable to the use of psychiatric medications such as selective serotonin reuptake inhibitors. Despite the fact that having a child born with a handicap is difficult for everyone concerned, little is known about the culture and experiences of men who have a child with a disability, since much prior study has concentrated on the mothers’ perspectives [27]. While studies often see dads as peripheral or external system members, the notion that the duties of fathers of children with disabilities are restricted to assisting the mother is being contested. Although not all fathers saw parenting a child with impairments as difficult, those who did assessed their children as less adaptive and acceptable, as well as more demanding, irritable, and distracted. In addition to greater melancholy and lower connection to their children, fathers with high stress perceived less competence as a parent, experienced social isolation, and had poor health outcomes [28].

Family stress theory-based research examining the experiences of parents parenting a child with a developmental handicap revealed that the child’s traits predicted just 8% of the stress experienced by fathers. The factors connected to family resources contributed to 33% of paternal stress, whereas the characterization and interpretation, of the issue, accounted for 37% [29]. The most significant predictor of parental stress was a negative situational description. Particularly for dads, this negative connotation was related to the child’s social acceptability. Thus, the observed views of other individuals affected the father’s assessment of an unhealthy child as a burden or disaster. To some authors, it seems that men were more affected than mothers by the unfavorable opinions of others and depended more on their spouse’s support and their own ability to cope [30].

Thus, parents of children with a chronic disease who are confronted with challenging parenting circumstances are less likely to seek emotional assistance from others, as described in the literature. They also seek moral support, friendship, compassion, and understanding less often than parents of healthy developing children [31]. This pattern would also result in a decrease in the intensity of avoidance methods since the primary emphasis is on childcare. These results are significant because they may explain the correlation between depression and social disengagement among parents of children with disabilities. Indeed, social support is a protective factor against emotional issues and poor self-perceived life satisfaction, and it might be a crucial aspect in the development of successful support programs for this population of parents. Fascinatingly, parents did not exhibit the major tactics associated with obtaining emotional support from religion, which are more prevalent among parents of normal children. In this regard, parents may see religious coping techniques as maladaptive. Other investigations, however, show that religious support is favorable. In this regard, the findings of prior research involving children with various types of disabilities are inconsistent [32].

Regarding coping techniques, our study identified the avoidance-oriented mechanism as the most prevalent among the affected fathers. This is in accordance with one research that found substantial differences between parents of children with developmental disabilities and parents of children without developmental disabilities for one of three coping strategies [33]. Less often did parents of children with developmental impairments use the avoidance-oriented and emotional support styles. The task-oriented attitude and tactics were prevalent across both parent groups. In stressful circumstances associated with childrearing, parents of children with developmental impairments do not rely on emotional support and religion as often as parents of healthy children.

### 4.2. Strengths and Limitations

Even though the present research complied with the very minimal standards for sample size requirements, there are some weak points of the study that need to be pointed out. First, the cases and control samples were not matched, therefore it was not possible to control for confounding factors. Thus, a matched sample could provide more accurate results. Second, the cross-sectional design might be considered a limiting feature since it does not provide a compelling evaluation and assessment in time of the stress levels and intimacy problems experienced by the men involved. This prevents the levels of stress and couple satisfaction from being determined in an accurate manner since the surveys measure only the participants’ answers at one point in time. As a third limitation, the use of questionnaires may result in a high subjectivity index from all of the participants who consented to fill them out, which can lead to several biases in the data collected. Another possible limitation of the study is the lack of a main outcome; therefore, causality cannot be determined from the studied survey scores as predictors. Further limitations comprise the lack of comparison with the maternal side, the bias of reporting intimacy and intimate questionnaire responses, as well as there being no experimental setting or controlled matching to interpret findings in a clearer manner. In conclusion, the findings of the present research can only be generalized to the population that was investigated because of the possibility that some unique aspects of the population, such as religion and culture, would have a particular effect on the findings.

## 5. Conclusions

According to the data that have been presented, it is probable that paternal stress is greater near children who have cystic fibrosis, and the absence of assistance in this vulnerable group seems to lead to difficulties with intimacy, couple dissatisfaction, and maladaptive coping methods. It is recommended that these concerns are not only raised for the mothers of children with mucoviscidosis but also for the child’s father or the male partner taking care of the child, as they might experience the same problems as the opposite gender. Considering the modern family structure, it is recommended that these concerns should not only be raised for the mothers of children with mucoviscidosis. Further studies might seek to develop parental and child interventions based on our findings, to reduce the burden of reported difficulties.

## Figures and Tables

**Table 1:** Comparison of background characteristics and behaviors between fathers with a disabled child (cases) and fathers with normal children (controls).

Variables	Cases (*n* = 107)	Controls (*n* = 124)	*p*-Value
Age, years (mean ± SD)	35.9 ± 9.4	34.6 ± 9.0	0.284
Age range (years)			0.620
<25	12 (11.2%)	17 (13.7%)	
25–35	40 (37.4%)	51 (41.4%)	
>35	55 (51.4%)	56 (45.2%)	
Area of Residence (urban)	66 (61.7%)	63 (50.8%)	0.096
Relationship Status			0.569
Married/Concubinage	98 (91.6%)	116 (93.5%)	
Single/Divorced/Widowed	9 (8.4%)	8 (6.5%)	
Income (medium and high)	71 (66.4%)	90 (72.6%)	0.304
Education (university degree)	39 (36.4%)	47 (37.9%)	0.819
Religion			0.737
Orthodox	86 (80.4%)	98 (79.0%)	
Catholic	6 (5.6%)	5 (4.0%)	
Protestant	7 (6.5%)	7 (5.6%)	
Not specified	8 (7.5%)	14 (11.3%)	
Practicing religion (yes)	53 (49.5%)	47 (37.9%)	0.075
Occupation (employed or self-employed)	72 (67.3%)	95 (76.6%)	0.114
Sexual activity			0.021
Weekly or more	24 (22.4%)	37 (29.8%)	
1–3 times per month	43 (40.2%)	61 (49.2%)	
Less than 1–3 times per month	40 (37.4%)	26 (21.0%)	
Substance use behavior			
Frequent alcohol consumption	9 (8.4%)	5 (4.0%)	0.164
Frequent smoker	37 (34.6%)	28 (22.6%)	0.043
Drug user	3 (2.8%)	3 (2.4%)	0.854

Data reported as *n* (frequency) and calculated using Chi-square test and Fisher’s exact unless specified differently.

**Table 2:** Comparison of unstandardized survey questions between cases and controls.

Variables	Cases (*n* = 107)	Controls (*n* = 124)	*p*-Value
Number of children			<0.001
1	95 (88.8%)	76 (61.3%)	
≥2	12 (11.2%)	48 (38.7%)	
Age of children			0.019
Infancy	59 (55.1%)	61 (49.2%)	
Childhood	37 (34.6%)	33 (26.6%)	
Adolescence	11 (10.3%)	30 (24.2%)	
Psychiatric disease (yes)	11 (10.3%)	7 (5.6%)	0.190
Marital distress (yes)	16 (15.0%)	8 (6.5%)	0.034
Attending psychotherapy (yes)	5 (4.7%)	1 (0.8%)	0.065
Attending couple therapy (yes)	7 (6.5%)	3 (2.4%)	0.124
Taking psychiatric medication (yes)	8 (7.5%)	2 (1.6%)	0.028
Comorbid conditions	23 (18.7%)	29 (23.4%)	0.384
Reproductive problems			
Erectile dysfunction	13 (12.1%)	10 (8.1%)	0.301
Ejaculation problems	9 (8.4%)	11 (8.9%)	0.901
Inhibited sexual desire	26 (24.3%)	17 (13.7%)	0.039

Data reported as *n* (frequency) and calculated using Chi-square test and Fisher’s exact unless specified differently; CF—Cystic Fibrosis.

**Table 3:** Comparison of CISS survey results between cases and controls.

Items (Score Range)	Cases (*n* = 107)	Controls (*n* = 124)	*p*-Value
Task-oriented (high)	33 (30.8%)	61 (49.2%)	0.004
Emotion-oriented (high)	27 (25.2%)	35 (28.2%)	0.608
Avoidance-oriented (high)	49 (45.8%)	32 (25.8%)	0.002

Data reported as *n* (frequency) and calculated using Chi-square test and Fisher’s exact unless specified differently. CISS—Coping Inventory for Stressful Situations.

**Table 4:** Comparison of PSI-SF survey results between cases and controls.

Components	Cases (*n* = 107)	Controls (*n* = 124)	*p*-Value
Parenting Distress (high)	46 (43.0%)	33 (26.6%)	0.008
Parent–Child Dysfunctional Interaction (high)	29 (27.1%)	30 (24.2%)	0.613
Difficult Child (high)	37 (34.6%)	23 (18.5%)	0.005
Total Stress (high)	44 (41.4%)	28 (22.6%)	0.002

Data reported as *n* (frequency) and calculated using Chi-square test and Fisher’s exact unless specified differently. PSI-SF—Parenting Stress Index.

**Table 5:** Comparison of SII and DSCS survey results between cases and controls.

Items	Cases (*n* = 107)	Controls (*n* = 124)	*p*-Value
Dissatisfaction	27.1 ± 2.6	23.4 ± 3.0	<0.001
Self-acceptance	8.5 ± 2.9	10.2 ± 3.3	<0.001
Pleasure	4.4 ± 2.7	4.9 ± 3.1	0.195
Perceptual accuracy	19.9 ± 4.3	17.8 ± 5.5	0.002
Mate acceptance (marital quality)	13.8 ± 1.9	16.5 ± 2.2	<0.001
SII total score	73.7 ± 14.4	72.8 ± 17.1	0.668
DSCS total score	42.1 ± 11.6	48.7 ± 9.9	<0.001

Data reported as mean ± SD and calculated using Student’s *t*-test; SII—Sexual Interaction Instrument; DSCS—Dyadic Sexual Communication Scale.

**Table 6:** Comparison of MIQ survey results between cases and controls.

Items	Cases (*n* = 107)	Controls (*n* = 124)	*p*-Value
Intimacy problems	37.2 ± 10.4	34.3 ± 8.6	0.021
Consensus	36.4 ± 7.7	33.6 ± 7.0	0.004
Openness	37.9 ± 7.0	39.9 ± 8.5	0.054
Affection	29.6 ± 5.8	30.2 ± 6.1	0.446
Commitment	32.3 ± 6.5	30.7 ± 5.7	0.047

Data reported as mean ± SD and calculated using Student’s *t*-test; MIQ—Marital Intimacy Questionnaire.

## Data Availability

The data presented in this study are available on request from the corresponding author.

## References

[1] Sanders D.B., Fink A.K. (2016). Background and Epidemiology. Pediatr. Clin. N. Am..

[2] Chen Q., Shen Y., Zheng J. (2021). A review of cystic fibrosis: Basic and clinical aspects. Anim. Models Exp. Med..

[3] McBennett K.A., Davis P.B., Konstan M.W. (2022). Increasing life expectancy in cystic fibrosis: Advances and challenges. Pediatr. Pulmonol..

[4] Keogh R.H., Szczesniak R., Taylor-Robinson D., Bilton D. (2018). Up-to-date and projected estimates of survival for people with cystic fibrosis using baseline characteristics: A longitudinal study using UK patient registry data. J. Cyst. Fibros..

[5] Durda-Masny M., Goździk-Spychalska J., John A., Czaiński W., Stróżewska W., Pawłowska N., Wlizło J., Batura-Gabryel H., Szwed A. (2021). The determinants of survival among adults with cystic fibrosis-a cohort study. J. Physiol. Anthropol..

[6] Yamaoka Y., Tamiya N., Moriyama Y., Sandoval Garrido F.A., Sumazaki R., Noguchi H. (2015). Mental Health of Parents as Caregivers of Children with Disabilities: Based on Japanese Nationwide Survey. PLoS ONE..

[7] Gurtovenko K., Fladeboe K.M., Galtieri L.R., King K., Friedman D., Compas B., Breiger D., Lengua L., Keim M., Kawamura J. (2021). Stress and psychological adjustment in caregivers of children with cancer. Health Psychol..

[8] Continisio G.I., Serra N., Guillari A., Civitella M.T., Sepe A., Simeone S., Gargiulo G., Toscano S., Esposito M.R., Raia V. (2020). An investigation on parenting stress of children with cystic fibrosis. Ital. J. Pediatr..

[9] Deffaa M., Weis M., Trommsdorff G. (2020). The Role of Maternal Parenting for Children’s Behavior Regulation in Environments of Risk. Front. Psychol..

[10] Sheidow A.J., Henry D.B., Tolan P.H., Strachan M.K. (2014). The Role of Stress Exposure and Family Functioning in Internalizing Outcomes of Urban Families. J. Child Fam. Stud..

[11] Lee D., McLanahan S. (2015). Family Structure Transitions and Child Development: Instability, Selection, and Population Heterogeneity. Am. Sociol. Rev..

[12] Jessee V., Adamsons K. (2018). Father Involvement and Father-Child Relationship Quality: An Intergenerational Perspective. Parent. Sci. Pract..

[13] Dong S., Dong Q., Chen H., Yang S. (2022). Mother’s Parenting Stress and Marital Satisfaction During the Parenting Period: Examining the Role of Depression, Solitude, and Time Alone. Front. Psychol..

[14] Farrell P.M. (2008). The prevalence of cystic fibrosis in the European Union. J. Cyst. Fibros..

[15] Endler N.S., Parker J.D. (1990). State and trait anxiety, depression and coping styles. Aust. J. Psychol..

[16] Spratt E.G., Friedenberg S.L., Swenson C.C., Larosa A., De Bellis M.D., Macias M.M., Summer A.P., Hulsey T.C., Runyan D.K., Brady K.T. (2012). The Effects of Early Neglect on Cognitive, Language, and Behavioral Functioning in Childhood. Psychology.

[17] LoPiccolo J., Steger J.C. (1974). The sexual interaction inventory: A new instrument for assessment of sexual dysfunction. Arch. Sex. Behav..

[18] Catania J.A. (1998). Dyadic sexual communication scale. Handbook of Sexuality-Related Measures.

[19] van den Broucke S., Vandereycken W., Vertommen H. (1995). Marital intimacy: Conceptualization and assessment. Clin. Psychol. Rev..

[20] IBM Corp (2020). IBM SPSS Statistics for Windows.

[21] Microsoft Corporation (2018). Microsoft Excel. https://office.microsoft.com/excel.

[22] Raju S.V., Jackson P.L., Courville C.A., McNicholas C.M., Sloane P.A., Sabbatini G., Tidwell S., Tang L.P., Liu B., Fortenberry J.A. (2013). Cigarette smoke induces systemic defects in cystic fibrosis transmembrane conductance regulator function. Am. J. Respir. Crit. Care Med..

[23] Darling C.A., Senatore N., Strachan J. (2012). Fathers of children with disabilities: Stress and life satisfaction. Stress Health.

[24] Siracusano M., Riccioni A., Gialloreti L.E., Segatori E., Arturi L., Vasta M., Porfirio M.C., Terribili M., Galasso C., Mazzone L. (2021). Parental Stress and Disability in Offspring: A Snapshot during the COVID-19 Pandemic. Brain Sci..

[25] Sarimski K. (2017). Erlebte Belastung von Müttern von Kindern mit Down-Syndrom im Vorschulalter [Parenting Stress in Mothers of Children with Down Syndrome in Preschool Age]. Prax. Kinderpsychol. Kinderpsychiatr..

[26] Kiernan J., Mitchell D., Stansfield J., Taylor C. (2019). Mothers’ perspectives on the lived experience of children with intellectual disability and challenging behaviour. J. Intellect. Disabil..

[27] Duchovic C.A., Gerkensmeyer J.E., Wu J. (2009). Factors associated with parental distress. J. Child Adolesc. Psychiatr. Nurs..

[28] Saloviita T., Itälinna M., Leinonen E. (2003). Explaining the parental stress of fathers and mothers caring for a child with intellectual disability: A Double ABCX Model. J. Intellect. Disabil. Res..

[29] Fucà E., Costanzo F., Ursumando L., Vicari S. (2022). Parenting Stress in Mothers of Children and Adolescents with Down Syndrome. J. Clin. Med..

[30] Ward K.P., Lee S.J. (2020). Mothers’ and Fathers’ Parenting Stress, Responsiveness, and Child Wellbeing Among Low-Income Families. Child Youth Serv. Rev..

[31] Vernhet C., Dellapiazza F., Blanc N., Cousson-Gélie F., Miot S., Roeyers H., Baghdadli A. (2019). Coping strategies of parents of children with autism spectrum disorder: A systematic review. Eur. Child Adolesc. Psychiatry.

[32] Shokoohi-Yekta M., Ghobary-Bonab B., Malayeri S.A., Zamani N., Pourkarimi J. (2015). The relationship between anger and coping strategies of mothers of children with special needs. Procedia-Soc. Behav. Sci..

[33] Bujnowska A.M., Rodríguez C., García T., Areces D., Marsh N.V. (2021). Coping with stress in parents of children with developmental disabilities. Int. J. Clin. Health Psychol..

